# An online course about cosmetics improves skin care practices and skin health

**DOI:** 10.3389/fpubh.2022.951481

**Published:** 2022-09-09

**Authors:** Yu Li, Wei Hua, Jie Tang, Lidan Xiong, Li Li

**Affiliations:** ^1^Center of Cosmetics Evaluation, West China Hospital, Sichuan University, Chengdu, China; ^2^Department of Dermatology, West China Hospital, Sichuan University, Chengdu, China; ^3^NMPA Key Laboratory for Human Evaluation and Big Data of Cosmetics, Chengdu, China; ^4^Sichuan Engineering Technology Research Center of Cosmetic, Chengdu, China

**Keywords:** massive open online course, cosmetics, skin care, education, public health promotion

## Abstract

**Background:**

The incidence of cosmetics-associated dermatoses is on the rise recently while the awareness and knowledge about proper utilization of cosmetics are insufficient in both the public and specialists due to limited education about cosmetics.

**Methods:**

Our aim was to build and enhance the public's ability to select appropriate cosmetics, manage possible dermatoses and improve skin-care practices and skin health by offering access to medical information *via* free online courses. Consequently, we launched a massive open online course (MOOC), Appreciation and Analysis of Cosmetics. An online questionnaire was also sent to evaluate the effectiveness of the course.

**Results:**

Nearly 540,000 learners were enrolled in the course since 2014. In the discussion forum, there were 8,383 posts and 73,014 replies in total, where learners were mostly concerned about topics of sun protection, cleansing and proper utilization of cosmetics in some skin diseases. 645 learners answered the questionnaire with 88.84% of the them changed skin care practices and 50.39% reported improvement in skin health. Moreover, participants who completed the course reported better understanding and utilization of cosmetics-related knowledge (*p* values < 0.05). 72.09% of respondents were willing to recommend this course to others.

**Conclusions:**

Free online public courses are feasible for conducting public health education campaigns related to cosmetics and associated dermatology to lower the incidence of cosmetics-associated dermatoses.

## Introduction

Cosmetics industry has shown continued development in recent years and cosmetics have gradually become a necessity in people's daily lives. It is noteworthy that cosmetic products have been proven to be closely associated with a series of sub-health skin conditions and diseases including impaired skin barrier, contact dermatitis, acne, rosacea, and so on. The proper utilization of these products can be highly beneficial to patients as an effective adjuvant treatment and a leading prevention strategy. On the contrary, inappropriate application, a phenomenon which bothers a significant number of consumers worldwide, will bring about skin diseases or worsen original dermatoses and dramatically lower consumers' quality of life (1–9). Besides, products containing illegal components, for instance, glucocorticoids and heavy metals, greatly undermined consumers' health, and in rare cases, even inducing kidney failure and other life-threatening diseases (10–12). Therefore, it is essential for consumers to use cosmetics properly and recognize cosmetic related adverse reactions. In the meanwhile, dermatologists should routinely inform patients to adopt proper procedures of applying cosmetics to enhance treatment effects and to avoid unsuitable or unqualified products. Unfortunately, in the practice, we found that the public and patients, even some dermatologists were lack of awareness or had incorrect cognition about cosmetics. One possible reason, from consumers' perspectives, is the lack of formal platform to gain cosmetic knowledge, which renders them confused when trying to decide which cosmetic products to purchase when confronted with eye-catching advertisements of diverse products. For doctors, the traditional education of dermatology pays little attention to cosmetics, which leads to very insufficient awareness of the proper utilization and the adverse reactions of cosmetics, that in fact, are extremely common in the clinic.

Fortunately, the online courses, an emerging pattern of education mode, may solve this problem. For instance, massive open online courses (MOOC) provides a free platform to the public to easily get access to thousands of professional courses with high teaching efficiency (13–15). In order to propose information about cosmetics and its related adverse reactions to the public, our team has designed and released an online course, *Appreciation and Analysis of Cosmetics*, in the platform of MOOC since 2014. The objective of this study is to evaluate the effectiveness of the open online course in improving the cosmetic knowledge and skin care practices and any subsequent changes in skin conditions of participants.

## Materials and methods

### Course design

#### Eligibility

The *Appreciation and Analysis of Cosmetics* course was launched in September 2014 on a website named MOOC of Chinese Universities, which was freely available to individuals worldwide. There would be two semesters each year. Participants were expected to spend about 50 min per week (total 15 weeks) engaging with the course content and assignments.

#### Course content

The course content comprised of three categories, which we have mentioned before (13). Briefly, the category one is about manufacture of cosmetics, the category two is functions of common cosmetics, 11 types of products with different shapes and functions, such as moisturizing, sunscreen, facial mask, perfume, etc., and category three is cosmetics-associated dermatology.

This 15-week course consisted of 15 lessons, which was divided into 3–5 videos lasting 10–15 min. Participants had the options of watching videos online or downloading them to portable devices; this ensured that learners were able to study whenever convenient and review specific content repeatedly at will. Corresponding illustrations and text explanations were also provided along with the videos as supplementary learning materials to enhance comprehensibility of the lessons. To keep participants' enthusiasm, examples of popular cosmetic products were also integrated into the curriculum; this aimed to ensure that the course content remained practically viable.

Coupled with each lesson, weekly homework assignments and quizzes were launched to further reinforce learning as well as enhance participants' practical application of course content. In addition, the website offered a forum for after-class discussions between learners and teaching staff, aiming to generate communication opportunities and emulate the atmosphere of a realistic classroom setting.

#### Examination

Participants' final grades reflected their performance in homework assignments (30%), quizzes (20%), the final exam (40%), and forum discussion activities (10%). With an aggregate score exceeding 60 points, participants were eligible for a “certificate of quality” and learners whose scores surpassed 85 qualified for a “certificate of excellence.”

#### Questionnaire

In the semester 10, to evaluate the impact of the course objectively, we sent a questionnaire *via* e-mail to participants to assess their comprehension of cosmetics-related issues. The questionnaire also asked participants if they had changed their skin care practices and whether they noticed improvements in their skin conditions after attending the course. Survey responses were collected by Wenjuanxing Website, a free online questionnaire platform. The options are worth 1–4 points respectively and data were analyzed using the Mann-Whitney *U*-test and Spearman's rank correlation with SPSS 22.0. *p* < 0.05 was considered statistically significant.

## Results

**Figure 1:**
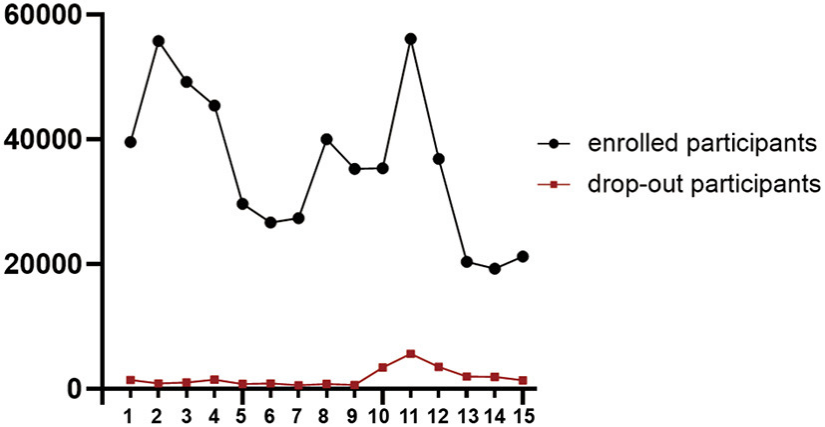
The number of enrolled and drop-out participants from 1 to 15 semesters.

To date, 15 semesters of our MOOC have been fully launched. Nearly 540,000 learners have been enrolled since 2014. The detailed information was shown in Table 1. The number of participants varied from years to years. And in the year of 2020, we launched the semester 11, which had the most learners (*N* = 56,133). The drop-out rate was 1.56–10.01% with an average of 4.87% (Figure 1). The number of learner taking the final exams reached to 7,333 in all. As for the pass rate, it has dramatic change from 2.97 to 27.59%. Moreover, there were 4,566 participants eligible for a “certificate of quality,” among them, 1,699 were qualified as excellent learners.

**Table 1:** Information about *Appreciation and Analysis of Cosmetics*.

**/Semester**	**1**	**2**	**3**	**4**	**5**	**6**	**7**	**8**	**9**	**10**	**11**	**12**	**13**	**14**	**15**	**All**
Enrollment	39,593	55,795	49,213	45,426	29,651	26,658	27,367	40,023	35,257	35,392	56,133	36,879	20,359	19,250	21,217	5,38,213
Drop-out (%)	3.59	1.56	2.05	3.27	2.60	3.27	2.13	1.89	1.81	9.63	10.01	9.54	9.65	9.91	6.43	4.87
Learners taking the final exam	465	322	109	195	379	299	891	422	633	416	1,214	1,347	346	103	192	7,333
Pass rate (%)	2.97	5.91	3.43	4.42	5.63	10.75	26.31	12.64	20.82	15.90	22.61	27.59	18.06	6.18	18.24	13.20
Qualified (score ≥60)	65	202	68	126	169	220	655	381	574	378	691	740	186	49	62	4,566
Excellent (score: 85–100)	7	10	7	11	11	26	35	83	188	212	436	520	88	33	22	1,699
** Scores in different categories**																
Manufactures (5)	4.55	4.64	4.62	4.72	4.55	4.61	4.55	4.55	4.64	4.6	4.55	4.55	4.47	4.13	4.33	4.57
Functions (5)	4.35	4.42	4.37	4.34	4.28	4.3	4.27	4.35	4.35	4.33	4.32	4.21	4.15	3.89	4.19	4.31
Cosmetic-associated dermatology (5)	5.00	4.9	4.9	5.00	4.9	4.9	4.8	4.9	4.9	4.9	4.8	4.8	4.9	4.9	4.7	4.87
Final exam (100)	69.95	73.49	72.66	70.9	70.25	74.18	77.54	77.61	76.77	79.96	87.21	93.52	85.56	87.35	81.64	81.35
** Discussion forum**																
Number of posts	1,164	779	507	826	1,565	581	547	437	762	446	412	258	31	28	40	8,383
Number of replies	7,191	3,486	2,172	3,539	11,009	2,447	3,286	2,523	5,613	6,048	9,433	8,925	3,578	1,204	2,560	73,014

In terms of three categories, the scores of functions of cosmetics were the lowest compared with the manufactures of cosmetics and cosmetic-associated dermatology (4.31 vs. 4.57, and 4.87, respectively). And the average score of final exam was 81.35.

In the discussion forum, there were 8,383 posts and 73,014 replies in total and in semesters 13–15, the number decreased sharply. Learners were extremely concerned about issues of sun protection, cleansing and how to choose cosmetics in skin diseases including acne vulgaris and sensitive skin.

Cronbach's α for the questionnaire responses was 0.857. 645 of the MOOC learners answered the questionnaire. The population characteristics were shown in Table 2. Significantly, 45.58% of the respondents completed the course while other 54.42% failed. Considering the time and reasons for dropping out, 29.80% respondents dropped out in the first month and the three most frequently mentioned reasons for quitting the course were “I didn't have enough time” (50.57%), “lack of practical use” (9.94%), and “the duration of the course was too long” (7.10%).

**Table 2:** Demographic information of respondents (*N* = 645).

	***n***	**%**
**Gender**		
Female	565	87.60
Male	80	12.40
**Age group**		
17–18	12	1.86
19–25	412	63.88
26–30	110	17.05
31–40	80	12.40
41–50	21	3.26
51–55	10	1.55
**Education**		
≤High school students	6	0.93
High school degree	22	3.41
Bachelor degree	484	75.04
≥Master degree	133	20.62
**Completed the course?**		
Yes	294	45.58
No	351	54.42
**If no, how long had you been engaged with the course before you gave up? (choose the most accurate option)**		
1 week	75	21.37
2 weeks	72	20.51
1 month	105	29.91
2 months	67	19.09
3 months	32	9.12
**Why did you give up?**		
I didn't have enough time	178	50.71
Lack of practice	35	9.97
Lack of atmosphere	22	6.27
Unappealing teaching methods	14	3.99
Lack of face-to-face discussion	14	3.99
The knowledge was too technical to understand	9	2.56
Other reasons	36	10.26

For participants who completed the course, the scores of every question and total course were all higher than those who failed to complete the MOOC course (Table 3, *p* < 0.05).

**Table 3:** The questionnaire about cosmetics-related knowledge before and after attending the course.

**Questions**	**Completed**		**Uncompleted**		***p-*Value**
	**Pre-course**	**Post-course**	**Pre-course**	**Post-course**	
**Can you distinguish between different skin types?**					
1. Absolutely not	16	1	35	12	0.021
2. Partially know how to distinguish, but sometimes judge incorrectly	134	35	175	114	
3. Partially know how to distinguish, and haven't misjudged so far	113	224	120	196	
4. Have enough knowledge and understanding and can make correct judgments	31	34	21	29	
**To which extent do you know about the types and functions of cosmetics used in daily life?**					
1. Absolutely no idea	33	1	46	18	<0.001
2. Roughly know the types but have no idea of their specific functions and risks	178	26	183	118	
3. Exactly know the types and roughly know specific functions and risks, but unable to use them in practice.	62	180	89	157	
4. Exactly know the types and specific functions, and able to use them in practice.	21	87	33	58	
**To which extent do you know about cosmetics ingredients?**					
1. Absolutely no idea	96	6	124	27	<0.001
2. Roughly know the types but have no idea of specific functions and risks	147	57	170	161	
3. Exactly know the types and roughly know specific functions and risks	40	181	50	135	
4. Exactly know the types and specific functions	11	50	7	28	
**Can you choose the right products for yourself?**					
1. Absolutely not	47	2	71	27	<0.001
2. Know which type of products suit me, but unable to choose the specific product	145	58	191	147	
3. Can pick up several candidates but cannot find out the best option	84	170	72	139	
4. Can choose the products that suit me most	18	64	17	38	
**Can you tell the truth or falsity of cosmetics advertisements?**					
1. Absolutely not	51	6	56	21	<0.001
2. Roughly know the definitions of what the advertisements advocate but have no idea of their truth or falsity	129	37	166	107	
3. Roughly know the mechanisms of what the advertisements advocate, but cannot determine whether the benefits of the product outweigh the price	89	183	112	172	
4. Exactly know the mechanisms and the actual value of what the advertisements advocate	25	68	17	51	
**Can you tell the truth or falsity of so called “cosmetics tips” in daily life?**					
1. Absolutely not	46	5	71	26	<0.001
2. Roughly know what the logic behind those tips is but I have no idea of their truth or falsity	141	56	166	133	
3. Can roughly judge the truth or falsity though I don't know the mechanisms well	88	164	97	157	
4. Exactly know the mechanisms work and can clearly judge the truth or falsity of such tips	19	69	17	35	
**To which extent have you changed your skin care practices after taking this course?**					
1. Almost no changes (<10%)	15		57		<0.001
2. Mild changes (10–30%)	104		183		
3. Moderate changes (30–50%)	135		94		
4. Most attitudes and habits have been changed (>50%)	40		17		
**To which extent has your skin condition improved after taking this course?**					
1. Getting worse	3		4		<0.001
2. Almost no changes	112		201		
3. Mild changes (<20%), such as getting smoother, white, soft or flexible	156		131		
4. Obvious positive changes (>20%)	23		15		
**To which extent do you want to recommend our course to others?**					
1. Never recommend it to anyone	3		11		0.001
2. It depends	63		103		
3. Might recommend it	91		109		
4. I will recommend it to everyone around me	137		128		

**Figure 2:**
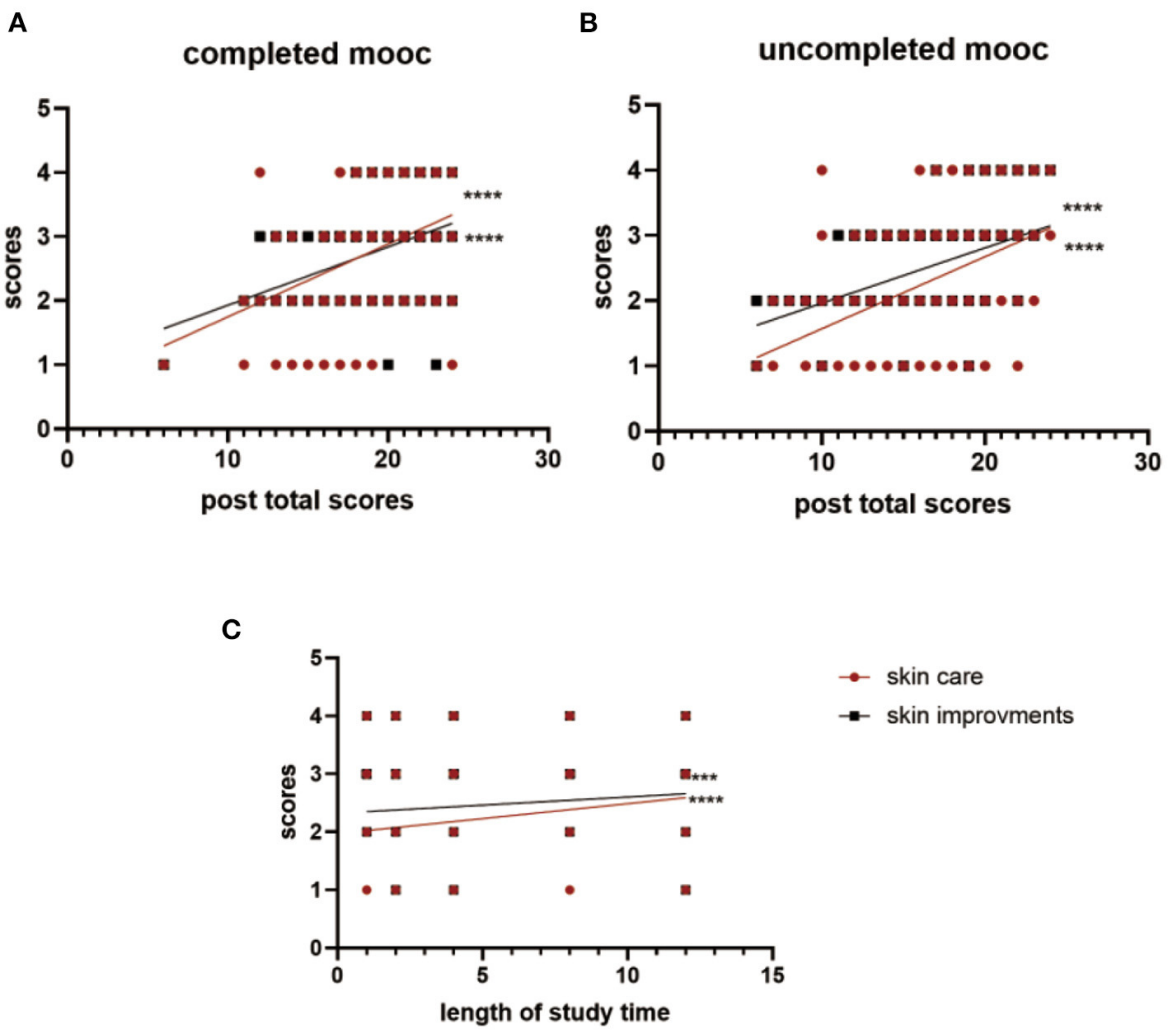
**(A)** The Spearman correlation co-efficients of completed MOOC participants' skin care practice changes and skin improvements correlated with their post total scores are 0.406 and 0.393 respectively, and the correlation co-efficient of the correlation between scores of skin care changes and skin improvements is 0.306 (*p* = 0.000). **(B)** The Spearman correlation co-efficients of uncompleted MOOC participants' skin care practice changes and skin improvements correlated with their post-total scores are 0.502 and 0.463 respectively, and the correlation co-efficient of the correlation between scores of skin care changes and skin improvements is 0.434 (*p* = 0.000). **(C)** The Spearman correlation co-efficients of uncompleted MOOC participants' skin care practice changes and skin improvements correlated with their length of study time are 0.258 and 0.202 respectively (*p* = 0.000). ***: *p* = 0.0001. ****: *p* < 0.0001. MOOC, massive open online course.

After the course, 88.84% of the learners changed their skin care practices, among them, 8.84% changed to a great extent. 50.39% the learners perceived moderate to apparent improvement in skin conditions. In both the completed and uncompleted groups, skin improvements were always positively correlated with post total scores and skin care practices changes (Figure 2). For those failing to complete the course, the time duration while they were engaged with the course also positively correlated with improvement of behavior changes and skin conditions. (ρ = 0.258, ρ = 0.202, respectively, *p* = 0.000), meanwhile, their pre-scores showed a positive correlation with post-scores (ρ = 0.648, *p* = 0.000).

As much as 72.09% of MOOC respondents were willing or strongly willing to introduce our course to people around them.

## Discussion

This study showed that a MOOC, *Appreciation and Analysis of Cosmetics*, was a novel effective approach to propagate valid skin care knowledge and educate the public in the appropriate selection of cosmetics and management of possible dermatoses. Most participants reported better understanding and utilization of cosmetics-related knowledge, including skin types, types, functions and risks of cosmetics, as well as the authenticity of advertising and rumors. Moreover, they reported positive changes in both skin care habits and skin conditions after the course.

The cosmetics industry is an emerging field with large market demand and widely-distributed audiences, which makes traditional teaching mode hard to meet the needs. In recent years, the rapid development of online courses have brought new opportunities toward it (14, 15). Furthermore, the traditional education of dermatology does not involve cosmetic-related courses, which hinders medical students and even dermatologists to recognize the importance of cosmetics for both normal skin and skin diseases. Thus, imparting cosmetics-related knowledge through online courses, can not only make up for the shortcomings of traditional teaching mode, but also enable students actively explore their interested information, and at the same time, provide the public with scientific cosmetics-related knowledge, thus to reduce the occurrence of cosmetics-related skin diseases.

*Appreciation and Analysis of Cosmetics*, composed of easily understandable short videos which conveyed detailed and applicable skin care information to audiences, functioned as a public health education resource for the ordinary populace. So far, almost 540,000 people have already been enrolled in this course since 2014. Unfortunately since 2021, in order to protect students' privacy, the platform of MOOC has no longer disclosed basic information of students. Thus, we can't get further general information about our participants such as gender, age, region and so on. But in our previous study, the results showed that most of the participants were female with a high level of education background and were from economically developed cities including Jiangsu, Zhejiang and Beijing (13).

The number of enrolled learners varied widely in different years. For example, in 2020, more learners were attracted to the course. We speculated the reason was that many people have more time to study online while isolating at home after the outbreak of the COVID-19. At the same time, some studies have revealed skin biophysical characters were changed after wearing masks, which may lead to or worsen skin diseases (16). People who suffered from these skin problems may join in this course to ask for help. However, as our teaching videos of MOOC was shot before 2014, the knowledge about cosmetics involved was relatively basic, thus many students reported the course content was difficult and lacked of practice, which may lead to the low completion rate.

In the three course categories, we found that learners' understanding of the functions of cosmetics were relatively lower than the other two categories. It is worth noting that we established courses in 11 sessions including moisturizers, oil-control, cleaning, sunscreen, whitening, allergy-relieving, anti-aging and hair-care products and perfumes, mask and make-up products. And learner were most confused in sunscreen and cleaning products, thus they raised many questions related in the discussion forum, such as “whether sunscreen is necessary on cloudy days,” “the choice of cleaning products,” and “how to choose such cosmetics under the skin disease state.” We found that these questions were common in the clinic as well, which indicated that the education of cosmetics and cosmetic-related dermatology are of great importance.

In order to evaluate the efficacy of our MOOC, we sent a questionnaire *via* e-mail to participants in the semester 10. A total of 645 learners responded to our survey. From the demographic data, it was not surprising that most participants of our MOOC were young female from coastal areas of China with good education background. Our previous study has showed that socioeconomic conditions and education have a significant impact on online learning, which was in line with this study (13).

Our study revealed nearly half of the respondents completed the course. They have better understanding and utilization of cosmetics-related knowledge than those who failed to complete the course, thus they have greater improvements in skin-care habits and skin condition. Though we took a series of measures to keep participants engaged, the dropout rate was still high. Compared with other MOOCs, which reported generally 10–20% completed rate, our MOOC has a considerably higher completed rate (17–19). The relatively long duration of the course combined with a lack of practical participation and regulation are the major causes for the high dropout rate. With detailed dropout analysis, we found that some students only learned courses of interest rather than the entire courses. It is worth noting that we think that identifying the skin type correctly is very important and basic, thus in the questionnaire, we established questions about distinction between different skin types as an assessment of students' knowledge about skin physiology. Despite the significant improvements in skin care knowledge and skin conditions of participants who completed the course, even participants who did not complete the full course demonstrated that the longer they remained engaged with the course, the more were able to benefit from it. Hence, our MOOC was proven to be a promising approach for bridging the knowledge gap between experts and the public. Nevertheless, this survey had some limitations. It was a retrospective study as we sent questionnaires to the participants after they finished the course, and thus they may have a bias in choosing the options. And people who responded to the questionnaire may have more enthusiasm for learning cosmetics than those ignored it.

To sum up, in view of teaching effectiveness and accessibility to general population, MOOCs merit the recognition of being dermatologists' preferred choice for conducting online public health intervention. A long-term follow-up is still required to observe the exact effects of our courses on lowering disease incidence and raising the quality of life. And in order to increase students' enthusiasm and sense of participation, thus improving the completion rate, we will re-plan and film the teaching contents and adopt online seminars to realize real-time interaction between teachers and students in the future, and further enhance the teaching effect of MOOC. In addition, shortening the period time of the course appropriately may also increase the learning engagement.

## Conclusions

As cosmetics-associated dermatoses have become a global public health issue, public health education and awareness on a large scale is becoming increasingly necessary. Our course, *Appreciation and Analysis of Cosmetics*, in the form of a MOOC, has proven to be a feasible approach to imparting knowledge about cosmetics and associated dermatology and improving skin care practices and skin conditions. Participants can benefit from the course even if they fail to complete it. Educating people in this manner could contribute to lowering incidence of cosmetics-associated dermatoses.

## Data availability statement

The original contributions presented in the study are included in the article/supplementary material, further inquiries can be directed to the corresponding author/s.

## Author contributions

LL and LX conceived the idea. YL and WH drafted the manuscript. LX and JT carried out revisions of the manuscript. LL finalized the manuscript. All authors contributed to drafting or revising the article, have agreed on the journal to which the article will be submitted, gave final approval of the version to be published, and agreed to be accountable for all aspects of the work.

## Funding

This work was supported by the 1.3.5 project for disciplines of excellence, West China Hospital, Sichuan University (ZY2016106).

## Conflict of interest

The authors declare that the research was conducted in the absence of any commercial or financial relationships that could be construed as a potential conflict of interest.

## Publisher's note

All claims expressed in this article are solely those of the authors and do not necessarily represent those of their affiliated organizations, or those of the publisher, the editors and the reviewers. Any product that may be evaluated in this article, or claim that may be made by its manufacturer, is not guaranteed or endorsed by the publisher.
